# Exploring patient views of empathic optimistic communication for osteoarthritis in primary care: a qualitative interview study using vignettes

**DOI:** 10.3399/BJGPO.2021.0014

**Published:** 2021-05-05

**Authors:** Emily Lyness, Jane Louise Vennik, Felicity L Bishop, Pranati Misurya, Jeremy Howick, Kirsten A Smith, Mohana Ratnapalan, Stephanie Hughes, Hajira Dambha-Miller, Jennifer Bostock, Leanne Morrison, Christian D Mallen, Lucy Yardley, Geraldine Leydon, Paul Little, Hazel Everitt

**Affiliations:** 1 GP and Academic Clinical Fellow, Primary Care, Population Sciences and Medical Education, University of Southampton, Southampton, UK; 2 Research Fellow, Primary Care, Population Sciences and Medical Education, University of Southampton, Southampton, UK; 3 Associate Professor in Health Psychology, School of Psychology, University of Southampton, Southampton, UK; 4 Research Assistant, Primary Care, Population Sciences and Medical Education, University of Southampton, Southampton, UK; 5 Senior Researcher and Impact Fellow, Department of Primary Care Health Sciences, University of Oxford, Oxford, UK; 6 Senior Research Assistant, Primary Care, Population Sciences and Medical Education, University of Southampton, Southampton, UK; 7 GP and Clinical Lecturer, Primary Care, Population Sciences and Medical Education, University of Southampton, Southampton, UK; 8 Patient Public Involvement Contributor, Southampton, UK; 9 Lecturer in Health Psychology, Primary Care, Population Sciences and Medical Education, University of Southampton, Southampton, UK; 10 Professor of General Practice and Public Health, Keele University, Keele, UK; 11 Professor in Health Psychology, University of Bristol, Bristol, UK; 12 Professor of Medical Sociology & Healthcare Interaction, Primary Care, Population Sciences and Medical Education, University of Southampton, Southampton, UK; 13 Professor of Primary Care Research, Primary Care, Population Sciences and Medical Education, University of Southampton, Southampton, UK

**Keywords:** physician–patient communication, physician–patient relations, placebo effect, primary health care

## Abstract

**Background:**

Osteoarthritis (OA) causes pain and disability. An empathic optimistic consultation approach can improve patient quality of life, satisfaction with care, and reduce pain. However, expressing empathic optimism may be overlooked in busy primary care consultations and there is limited understanding of patients’ views about this approach.

**Aim:**

To explore patients’ perspectives on clinician communication of empathy and optimism in primary care OA consultations.

**Design & setting:**

Vignette study with qualitative semi-structured interviews. Purposefully sampled patients (*n* = 33) aged >45 years with hip or knee OA from GP practices in Wessex (Hampshire, Dorest, Wiltshire, and Somerset).

**Method:**

Fifteen participants watched two filmed OA consultations with a GP, and 18 participants read two case vignettes. In both formats, one GP depicted an empathic optimistic approach and one GP had a ‘neutral’ approach. Semi-structured interviews were conducted with all participants and analysed using thematic analysis.

**Results:**

Patients recognised that empathic communication enhanced interactions, helping to engender a sense of trust in their clinician. They felt it was acceptable for GPs to convey optimism only if it was realistic, personalised, and embedded within an empathic consultation. Discussing patients’ experiences and views with them, and conveying an accurate understanding of these experiences improves the credibility of optimistic messages.

**Conclusion:**

Patients value communication with empathy and optimism, but it requires a fine balance to ensure messages remain realistic and trustworthy. Increased use of a realistic optimistic approach within an empathic consultation could enhance consultations for OA and other chronic conditions, and improve patient outcomes. Digital training to help GPs implement these findings is being developed.

## How this fits

Previous studies in trial and/or laboratory settings suggest that clinician communication with empathy and optimism is likely to improve patient satisfaction, and experience of pain via mechanisms underpinning contextual or ‘placebo’ effects. There is a paucity of evidence about how patients feel about this approach, and, therefore, how it might work in real-life primary care consultations. This study highlights that a fine balance is necessary to communicate realistic empathic optimism so that it is acceptable to patients. Clinicians who adopt this approach have the potential to enhance primary care consultations for OA and other chronic conditions, and improve patient outcomes.

## Introduction

Effective communication in primary care is vital to meet the complex needs of patients with chronic diseases and engage them with self-management of symptoms.^1–3^ There is a wealth of research into consultation styles, contributing to 'effective communication', with empathic verbal and non-verbal communication shown to have patient benefits including improved satisfaction and reduced pain.^1,4^ Showing empathy in the consultation, in combination with communicating optimistic expectations about treatment, has the potential to improve clinical outcomes via mechanisms underpinning contextual or ‘placebo’ effects.^3,5,6^ By conveying a positive message, clinicians can encourage patients to have positive outcome expectancies, that is, to believe a specific course of action or treatment will likely result in successful management of disease.^3,7^

Contextual and placebo factors make clinically meaningful contributions across diverse conditions including OA.^8–11^ This common and disabling condition affects one in five adults aged >45 years in the UK, with hip and knee being frequently affected joints.^12,13^ National Institute for Health and Care Excellence (NICE) guidance^12^ recommends non-pharmacological treatments such as exercise and weight loss first line, and these have a similar effect size to medications such as non-steroidal anti-inflammatory drugs.^14^ However, patients often seem reluctant to engage with or struggle to undertake these strategies,^15^ thus the need for communication approaches within primary care consultations that can better engage patients with these treatments.^16,17^ The benefits of empathic and optimistic communication are accepted in the literature,^4,18,19^ with evidence to show clinician communication style influences patient engagement with, and expectations of, positive health outcomes, which in turn can lead to improved patient outcomes such as reduced pain.^19,20^

Despite the growing evidence base, a combination of empathic and optimistic communication within primary care consultations is a relatively novel focus, and may not be currently prioritised in real-life busy consultations (Hughes *et al*, unpublished data, 2020). Much of the evidence on optimistic communication has been conducted in trial and/or laboratory settings without nested qualitative work,^3,7,19^ thus information is lacking on patients’ perspectives on the use of empathy and optimism in everyday clinical practice.

This study explores patients’ understanding and perspectives on empathic and optimistic communication in primary care consultations for OA, using a vignette approach to help elicit patients’ views.

## Method

The research was reported using the Standards for Reporting Qualitative Research framework.^21^

### Setting and participants

Patients, aged >45 years (NICE definition^12^) with a diagnosis of hip or knee OA, were recruited from three Wessex Clinical Research Network GP practices representing a range of demographic regions, urban and rural (see Supplementary Table S1). Patients were identified through an electronic medical records search for coded diagnosis including OA of the hip, knee, lower limbs, and ‘non-otherwise specified’. Patients who lacked capacity or did not speak English were excluded, along with those with a visual impairment preventing them from accessing vignette materials. All potential participants were eligibility screened by a practice GP, and sent invitation letters and patient information sheets. Interested patients returned a demographics questionnaire to the research team. Participants were purposefully sampled to ensure a range of demographics (age, ethnic group, sex, duration of OA symptoms, and history of joint replacement surgery), educational attainment, and deprivation index (see Supplementary Table S1). Semi-structured interviews were conducted face to face at the participant’s home, or GP surgery, allowing a comfortable, familiar environment. Participants provided written informed consent before the start of the interview.

### Study design

A qualitative interview study was conducted using fictional vignettes to selectively stimulate conversations about empathic and optimistic communication in GP consultations. One group of participants (*n* = 15) viewed two short films of OA consultations with actors (see Supplementary Box S1). The other group of participants (*n* = 18) read two written vignettes describing OA consultations. One film and one written vignette depicted an empathic, optimistic approach ('Dr Jones'); one film and one written vignette depicted a neutral approach ('Dr Smith') (see Supplementary Box S2). The vignette approach allowed standardised examples of an OA consultation to demonstrate these two communication approaches. Filmed examples helped demonstrate and elicit responses on both verbal and non-verbal communication behaviours under investigation. However, visual and auditory aspects of films can be distracting and lack relatability if elements differ from patients’ experiences. Participants’ reflections on written vignettes removed this issue and allowed participants to put themselves into the position of the patient, facilitating their interpretation of the fictional scenarios.^22–25^

Current literature on communication in primary care OA consultations was used to design the structure and content of the vignettes. The optimistic consultation script was informed by Howick *et al,* ^26^ which reported an analysis of positive message components used in 22 randomised trials of interventions seeking to optimise patients’ expectations. Neutral alternatives to these components were devised by the research team of clinicians and health psychologists, and were included in the control consultation to provide comparison. A panel of experts, including GPs and patient and public involvement (PPI) representatives, co-developed and agreed both vignettes to aid external validity.^24,27^

### Data collection

One author conducted semi-structured interviews with participants after watching the filmed consultations. Another author interviewed participants who had read the written vignettes. All participants were unknown to the interviewers.

The research team, including PPI representatives, developed two topic guides (see Supplementary Boxes S3 and S4) with similar broad questions to guide discussion around the vignettes. Topic guides and vignettes were piloted with PPI representatives before implementation to ensure both were understandable by patients. The topic guides developed iteratively as interviews progressed, informed by field notes and team discussion. This ensured assumptions or possible influences were identified and reflected on.

Sampling continued until the research team determined no further themes could be identified from the data and that further interviews were unlikely to elicit new insights that would alter main themes.^27–29^ Patients who met inclusion criteria but were not selected (*n* = 9) were thanked for their interest. All interviews were audiorecorded (30–60 minutes), transcribed verbatim, and anonymised.

### Analysis

A reflexive thematic analysis provided a flexible yet rich and detailed account of the data.^27–29^ The six phases of thematic analysis, as outlined by Braun and Clarke,^27^ were applied starting with familiarisation of the transcripts before coding in NVivo (version 11) software to allow iterative development of the coding manual. Interviews focused on the filmed vignettes and written vignettes were coded separately, grounding the search for patterns within the individual datasets (see Supplementary Table S2). Further synthesis was achieved through mapping these codes and creating subthemes that allowed an interpretation that draws together the complementary and contrasting patterns in the data (see Supplementary Table S3). Final themes were agreed and defined in discussion with the team, including clinicians, PPI representatives, and specialists in communication and placebo research. A summary of the findings were sent to interested participants. Themes were compared by participant age, sex, and OA joint affected, but no patterns were found (see Supplementary Table S1).

## Results

### Summary of themes

Three broad themes were developed, capturing patients’ views on the role of empathy and optimism in the primary care consultation. Theme 1: 'empathy engenders trust', describes the way clinician verbal and non-verbal empathic communication influences patients’ sense of trust and is instrumental in establishing a consultation that patients are more likely to be satisfied with. Theme 2: 'optimism engages patients', presents participants’ views on optimistic components of communication and how this affects patients’ expectations about their health outcomes. Theme 3: 'realistic optimism', highlights the fine balance necessary to convey empathic optimism in a realistic way that enhances the believability of clinicians’ health messages.

#### Theme 1: empathy engenders trust

##### Conveying empathy engenders a sense of trust in the clinician and enhances primary care consultations

Participants drew on their experience of healthcare interactions to describe key aspects of professionalism that they felt would help them trust their primary care clinician. These included the clinician appearing knowledgeable, empathic, kind, and respectful. Those viewing the filmed vignettes reflected on how they might feel if they were the patient in the example comparing this to the neutral vignette:

*'* *She was friendly, respectful and didn’t make you feel as though she was somebody up on a pedestal* *…* *you felt you could talk to her* *.'* (Participant [P]5, filmed vignette, about the empathic optimistic GP)

Participants identified both verbal and non-verbal components of the communication style depicted in the vignettes that would contribute to their satisfaction with the consultation. They picked out specific aspects of the GP’s attitude and demeanour in the written and filmed vignettes such as showing respect, patience, and kindness:

*'* *Well she was clearly welcoming right from the start to make him feel comfortable and she was attentive, made good eye contact and her body language showed that she wasn’t, you know, defensive.* *'* (P14, filmed vignette, about the empathic optimistic GP)

*'* *You’re going to come out of there thinking, I’ve been listened to intently, I’ve been given some solid advice from somebody who obviously knows what they’re talking about because their whole body structure, the way they spoke, the way they acted says that, and you’re going to believe it.* *'* (P18, written vignette, about the empathic optimistic GP)

Participants described how they recognise when a clinician is actively listening to them through being attentive. Participants watching the filmed vignettes commented on gestures such as the GP facing the patient, leaning forward, and maintaining eye contact while the patient spoke. In comparing these actions to those exhibited by the neutral GP, participants felt these actions would help to demonstrate that a clinician is interested in them as an individual:

*'* *She was obviously showing concern for the patient, she was leaning forward in the chair to sort of interact with him* *…* *she was really listening, that’s what came across.* *'* (P12, filmed vignette, about the empathic optimistic GP)

While they might prefer a personal history with a clinician, participants recognised that this continuity might not be possible in current general practice. Instead, they felt that demonstrating a holistic knowledge of patients through reference to their medical notes, or engaging in discussion about lifestyle, hobbies, and goals would help personalise the consultation:

*'* *I think they need to find out and build in your own health and history* *… there could be things that are linked to your personal history that will have a bearing on the osteoarthritis and your lifestyle* *…* *and that has to be taken into account.* *'* (P17, filmed vignette, about their own experience)

*'* *I would say that there tends to be an inconsistency of who you see* *… you tend to have to, more or less say the same thing to different people but I think once it’s there* [in the notes] *then they can access it* *.'* (P12, written vignette, about their own experience)

The GP in the empathic optimistic vignettes used approaches such as summarising concerns and acknowledging individual frustrations, which are absent from the neutral comparison. Participants highlighted that this demonstrates that a clinician understands patient perspectives, appreciates their concerns, and is genuinely interested in helping them. Participants identified that this validation makes them feel more engaged with their health problem, and, therefore, more likely to participate in the proposed management plan:

*'* *To be honest, if I was to see Dr Smith* [neutral GP] *I would come out maybe feeling a little bit disappointed, this one* [Dr Jones, empathic optimistic GP] *I would come out thinking yeah this is it. I would be feeling good mentally* *… knowing that okay* *… I’ve got all of these exercises to do, I’m going to do this then we’ll see what it’s like after two months* *… then fine and if not, there’s another one or two options* *.'* (P18, written vignette, about the empathic optimistic GP)

*'* *It makes you feel more comfortable with them, if they treat you the way Dr Jones* [empathic optimistic GP] *did, in my opinion you’ll leave the surgery feeling happier with yourself because he just seemed to be more concerned about you compared to Dr Smith* [neutral GP]*.'* (P4, written vignette, about the empathic optimistic GP)

#### Theme 2: optimism engages patients

##### Including optimistic components in the consultation supports patients to feel more positive about their health and engages them with the healthcare message

When discussing components of the communication demonstrated in the vignettes, participants expressed preference for hearing an individualised explanation for their symptoms, and a rationale for how the proposed management plan would benefit them. While they recognised some patients might be overwhelmed with in-depth descriptions, having an opportunity to request an explanation allowed them to feel empowered to manage their own symptoms. Participants felt that this engaged them and facilitated the delivery of messages about positive outcome expectations throughout the consultation:

*'* *It would make me feel more confident in the actual medication and what it’s supposed to do. The more information I think you can be given about* [the benefits of] *medicine the better. I know some people say “don't over overload me with science”, I feel the more information you can take on board, it makes you understand what’s happening to your body.* *'* (P1, written vignette, about the empathic optimistic GP)

Participants identified that positively reinforcing the message is valuable to consolidate information. They felt this confirmed the trustworthiness of the message about outcomes and could help to frame the process of self-management in an optimistic manner. When the empathic optimistic GP referenced their own experience, or the experience of similar patients, participants felt reassured to hear a similar self-management story with a successful outcome. They felt this would also give them confidence that the clinician is qualified to treat their condition. However, some highlighted the importance of the individualisation of care, and they wanted to know that advice and treatment suitable for another patient is relevant and appropriate for them:

*'* *Yes, it’s great* *—* *she’s done it for other people* *…* *virtually the exact same thing* *…* *this is very manageable, done it before; these are the good outcomes* *…* *most people go on and live a lovely life. Actually, it’s exactly that type of thing that is very reassuring when you have no idea what you're looking at.* *'* (P1, filmed vignette, about the empathic optimistic GP)

Participants recognised the value of clinicians keeping up to date with current evidence, and referencing guidelines helped to positively reinforce the believability of the healthcare message. However, some felt strongly that evidence needs to be personalised to individuals’ situations to make it appear less generic:

*'* *You’re making the same recommendation to me and the other person, right? But you’re also saying our evidence shows if you do this, then this is the benefit* *…* *in reality you’re not too interested who the other people are, but it’s the evidence that says, we know if you do this there’s the improvement for you.* *'* (P14, written vignette, about the empathic optimistic GP)

Participants noticed the optimistic framing of the management options and expected outcomes in the empathic optimistic vignettes. Most agreed that the use of positive and clear language to convey the likelihood of symptoms improving also helped motivate patients to feel more willing to try suggested treatments:

*'* *The way that she was presenting what she wanted him to do was very positive, this was what he had to do, and results would happen. I think that she was positive on that for trying to encourage him to do what she wanted to get the outcome he wanted.* *'* (P10, filmed vignette, about the empathic optimistic GP)

The neutral vignettes contained commonly heard terminology such as ‘wear and tear’ or associations of OA with ‘old age’. Participants reflected on this similarity with their own experience and that this painted a particularly negative outlook. They, therefore, welcomed the use of purposefully optimistic language when talking about OA. Participants also felt that positive language might directly influence patient wellbeing, making them feel happier and less anxious about their health. Participants felt this was particularly useful towards the end of the consultation as it influenced how they felt when they left the room:

*'* *If you’re feeling happier then you can deal with your pain better than if you come out feeling like “oh, they don’t really care”, so I think you’ll come away and sort of feel more on top of the world. I think frame of mind can help it, can make you feel better.* *'* (P3, written vignette, about the empathic optimistic GP)

Many participants were, however, cautious about the use of purposefully optimistic language in certain healthcare contexts including, for example, cancer. Despite an empathic approach to conveying this optimism, some participants were concerned that optimistic messages in some circumstances may not always be realistic and could be perceived to be disingenuous:

*‘I would probably be more doubtful even if you used* [positive] *phrases like that … it might be part of a dismissive procedure … I could see that they can be used in two different manners … as a ‘cudgel’ or as an encouragement.’* (P4, written vignette, about the empathic optimistic GP)

Participants were keen to share how their previous experience of living with OA and its management has shaped their understanding and perspectives on their health. Scepticism around the optimistic approach was identified when messages were seen as potentially lacking credibility, particularly if it conflicted with a patient’s well-established understanding of their condition:

*‘Well I wouldn’t be too much in his favour …* [Dr Jones] *is saying yeah it will make you better, but you think — well will it? It's been so long I’ve been having the problems and he is saying “well all this will make you better”, but nobody else has told me that before.’* (P5, written vignette, about the empathic optimistic GP)

#### Theme 3: realistic optimism

##### Achieving the balance of realistic optimism enhances the believability of the clinician recommendation

Participants recognised that individual patients have different ideas and expectations about how OA symptoms should be managed. They agreed that aligning an optimistic healthcare message with patients’ perspectives would be important to avoid this potential conflict. This would also reassure them about any misunderstanding and facilitate communication of a message they are more likely to believe:

*'* *When he made a point as to what he was doing, she responded to that and expanded on it* *…* *that he wanted to walk the dog. She picked up on that and said “as regards walking the dog, that’s a good thing, exercise is good”.* *'* (P2, filmed vignette, about the empathic optimistic GP)

The GP in the empathic optimistic vignette portrayed getting to know and connect with patients, and subsequently giving specific instructions for an individualised self-management plan. Participants felt this helped the optimistic message feel more realistic and the suggested outcomes more achievable:

*'* *She was listening to what he was saying. She showed, by repeating some of it back that she'd taken on board what he was saying. I think that was good* *…* *because he was already exercising a bit, and that was one thing that she was encouraging him* *…* *that what he already did is good, and that even the walking the dog, that is good for you.* *'* (P4, filmed vignette, about the empathic optimistic GP)

*'* *I liked the way that she gave him a timeframe, 6* *–* *8 weeks, so he’s not expecting a quick fix within the next 2. She’s made him understand or I feel she’s made the point of trying to make him understand that it’s not going to be immediate, but it will take time and the exercises; the leg will grow stronger if you do them.* *'* (P4, filmed vignette, about the empathic optimistic GP)

Participants highlighted the value of providing a specific timeframe for follow-up as the GP in the empathic optimistic vignette did. They thought this would reinforce the accountability of patients to engage with the management plan and felt that the clinician was giving them permission to manage their own symptoms. This respected their autonomy, but with the clinician still involved and invested in the outcome. It also acted as a safety net that they would be seen again if necessary. Participants found this reassuring and considered it an effective way of making the plan feel realistic for them to achieve:

*'* *I’d probably come out feeling somewhat refreshed* *…* *I feel good about this! It’s alleviated a few fears* *…* *yeah, I would come out feeling quite buoyant knowing that okay I’m going to get on and do these exercises* *…* *and see what the score is.* *'* (P18, written vignette, about the empathic optimistic GP)

## Discussion

### Summary

This study addressed the paucity of qualitative evidence about patients’ perspectives on the use of empathy and optimism in consultations.^30^ The analysis identified three key themes, which may guide clinicians to successfully apply an empathic optimistic approach in primary care consultations in a way that is acceptable to patients: empathy engenders trust; optimism engages patients; and realistic optimism.

Clinicians need to consider the impact of verbal and non-verbal communication behaviours on the credibility of communicated healthcare messages about expectations. Engendering trust is key to facilitate the communication of optimistic healthcare messages and to, thus, encourage patient engagement with self-management. Patients welcome framing information about OA in a positive way with a personalised explanation that is backed-up with evidence. Using purposefully positive language in the consultation can be acceptable; however, it must be realistic and relevant to avoid conflict with previously held beliefs about health. Time constraints and managing multiple health problems could make this approach tricky to achieve in real-life consultations. An approach incorporating both empathy and optimism requires a fine balance, but can facilitate the communication of healthcare messages that patients believe and are likely to respond to by engaging in their long-term symptom management.

### Strengths and limitations

A rigorous qualitative approach has allowed the collection of in-depth data from patients who have experience of living with OA, and consulting about management.^27,29^ The use of vignette case studies to stimulate discussion in the interviews enabled researchers to tackle the complexity of the concept from two angles. Asking participants to view consultations allowed them to imagine themselves in the position of the patient in the film and reflect on the non-verbal behaviours they witnessed. Reading the written consultations encouraged participants to share their own experience in comparison with the patient in the vignette.^23,24,29,31^ These approaches were, thus, mutually complementary, and have provided a breadth of data, not only on individual perspectives, but also the contexts in which these experiences are situated.^24,32^ This enhances the trustworthiness of the data collected. However, the use of vignettes required patients to imagine themselves in the described situations; this is not the same as patients actually experiencing empathic optimistic communication. Future work could involve clinicians implementing this approach to observe and analyse how this functions, and elicit patients’ views after they have personally experienced it.

Purposive sampling allowed recruitment of participants with varied demographic characteristics, including levels of educational attainment and from areas with different deprivation levels as a proxy of socioeconomic status.^29^ Health literacy measures were not collected. It is possible (but there is no strong evidence for it)^23,31^ that socioeconomic status and health literacy levels will influence communication preference; however, the present study found consistent themes across participants. Patient ethnicity may influence likelihood of trusting a clinician;^31^ unfortunately, the sample did not include participants from diverse ethnic backgrounds. Despite the heterogeneity of educational attainment of this sample, the complexities of a clinician’s communication style was a challenging concept for some participants. Owing to the iterative design of the topic guide, the level of enquiry was able to be adjusted to maximise participant understanding. The multidisciplinary research team, including PPI input into the design and analysis, enhanced the rigour of the work and the credibility of the findings.

### Comparison with existing literature

Trial and laboratory studies have identified that clinician conveyed optimism influences patient expectation.^3,8,19^ The data from the present study agrees with this, but highlights the possible pitfalls of this approach in real-life contexts.

Participants in this study felt that optimistic communication behaviours are likely to be better received if they are conveyed in a way that acknowledges and validates patients’ experiences. These findings triangulate with data from observational studies about the impact of empathic communication in the consultation.^33,34^ This work also highlights the importance of demonstrating an interest in the patient’s life^33,34^ and aligns with literature recommending patients be seen as experts in their own health.^35^

Clinician awareness is required throughout the consultation to remain alert to the patient’s perspective, which may differ from that of the clinician. It is recognised that clinicians and patients often have different yet similarly rational perspectives on the same subject, which can create a barrier to effective consultations, particularly in chronic conditions.^15,36,37^ Getting to know patients in a holistic way can ensure recommendations about management are realistic and designed to take into account the needs of the individual. However, participants acknowledged that this can be difficult to achieve in time-pressured consultations (Hughes *et al*, unpublished data, 2020). Sensitivity to the patient’s perspective confirms to them that they have been listened to and can make them feel more engaged in the consultation.^36,37^

This research also highlights the importance of avoiding the communication of unrealistic optimism and the risk of invoking the nocebo effect, a phenomenon during an encounter that can do harm as well as good.^9,37^ Participants highlighted that unrealistic optimism could disengage them from self-management of symptoms. This potential pitfall chimes with the findings of a recent review^38^ into the role of placebo in pain management. This identified an alignment in belief and desire for treatment outcomes between patient and clinician as a key factor for successful enhancement of contextual or placebo responses. Conveying optimism is, therefore, only likely to optimise outcomes if the consultation is tailored to the individual perspectives of the patient, and they believe and trust the clinician.

### Implications for research and practice

To the authors’ knowledge, this is the first qualitative attempt to identify patients’ perspectives on the combined use of empathy and optimism to enhance engagement and patient expectations about OA outcomes in primary care. This study highlights the importance to patients of clinicians employing empathetic communication to engender trust, therefore laying the groundwork for the use of realistic optimism in the consultation. Patients felt that increased use of this approach had the potential to enhance primary care consultations for OA, and other chronic conditions, by improving patient engagement with treatment and expectations about health outcomes. These findings are unlikely to be unique to OA or primary care, and patients’ views should be sought in different contexts (that is, other diseases in other healthcare settings) and with other patient groups (that is, ethnic minorities and younger age groups).

This communication approach should be incorporated into communication skills training for GP registrars and as continuing professional development opportunities for practising GPs wishing to enhance their consultation style. This study has informed the development of the Empathica communication tool for this purpose, which is currently undergoing feasibility testing in a National Institute for Health Research School for Primary Care Research-funded study (https://www.southampton.ac.uk/medicine/academic_units/projects/empathica.page).

Patients value communication with empathy and optimism, but this requires a fine balance to ensure messages remain both realistic and trustworthy. Increased use of realistic optimism within an empathic consultation could enhance consultations for OA and other chronic conditions, and improve patient outcomes.
